# Design of κ-Opioid Receptor Agonists for the Development of Potential Treatments of Pain with Reduced Side Effects

**DOI:** 10.3390/molecules28010346

**Published:** 2023-01-01

**Authors:** Federica Santino, Luca Gentilucci

**Affiliations:** Department of Chemistry “G. Ciamician”, University of Bologna, Via Selmi 2, 40126 Bologna, Italy

**Keywords:** κ-opioid receptor, biased agonist, partial agonist, antinociception, dysphoria, mood disorders, molecular docking, peptidomimetic, peripheral analgesia

## Abstract

The κ-opioid receptor (KOR) has recently emerged as an alternative therapeutic target for the development of pain medications, without deleterious side effects associated with the μ-opioid receptor (MOR). However, modulation of KOR is currently under investigation for the treatment of depression, mood disorders, psychiatric comorbidity, and specific drug addictions. However, KOR agonists also trigger adverse effects including sedation, dysphoria, and hallucinations. In this respect, there is currently much debate on alternative paradigms. Recent effort has been devoted in search of biased ligands capable of selectively activating favorable signaling over signaling associated with unwanted side effects. On the other hand, the use of partial agonists is expected to allow the analgesia to be produced at dosages lower than those required to produce the adverse effects. More empirically, the unwanted central effects can be also avoided by using peripherally restricted agonists. In this review, we discuss the more recent trends in the design of KOR-selective, biased or partial, and finally, peripherally acting agonists. Special emphasis is given on the discussion of the most recent approaches for controlling functional selectivity of KOR-specific ligands.

## 1. Introduction

The κ-opioid receptor (KOR) belongs to the class of inhibitory GPCRs, widely expressed throughout the CNS and peripheral tissues [1,2]. Together with the μ-, δ- and the nociceptin-opioid receptors (MOR, DOR, NOR) [3], KOR is involved in modulation of pain, reward, mood state, and cognitive functions [4,5]. The endogenous ligands of KOR are the Dynorphins [6], a family of neuropeptides of varying lengths that are formed from the precursor prodynorphin. KOR activation elicits potent antinociceptive effects in various models of acute (mechanical, thermal, and chemical), inflammatory, neuropathic, and cancer pain.

At present, the large majority of therapeutic painkillers are naturally occurring opiate MOR agonists, such as morphine, codeine, or semi-synthetic opioid derivatives, e.g., oxycodone, or fully synthetic molecules such as fentanyl or tramadol. On the other hand, the use of these drugs is accompanied by severe side effects, i.e., addiction and high abuse potential, slowed breathing, constipation, nausea, confusion, and drowsiness. Agonist activity on DOR does not lead to the same adverse effects associated with MOR agonists, but DOR agonists were found to display proconvulsive activity. Interestingly, the analgesic effects mediated by NOR are more complex than those elicited by the other opioid receptors. Indeed, agonism at NOR was shown to produce either anti- or pro-nociceptive effects depending on the route of administration and dosage [3].

Due to the implications of KOR activation, KOR agonists have attracted recent attention for their ability to produce potent analgesic effects without the harmful side effects typically associated with MOR activation [7,8]. KOR agonists present low abuse potential and may also prevent hyperalgesia produced by chronic use of MOR-targeting drugs [9]. In addition to analgesia, KOR agonists showed potential for the treatment of pruritis, multiple sclerosis, Alzheimer’s disease, immune mediated diseases such as osteoarthritis, atopic dermatitis, food allergy, gastrointestinal (GIT) diseases, cancer, and hypoxia and ischemia [9].

Despite their potential efficacy, at present no KOR agonist is used to treat pain in humans, mostly due to relevant side effects, i.e., severe dysphoria, neuropathy-induced astrocyte proliferation and subsequent hyperalgesia, sedation, coordination impairment, and anhedonia. Albeit not exploited as analgesics, the many selective KOR agonists discovered so far have aroused much attention for their potential to treat abuse conditions, in particular those of cocaine or alcohol [10]. In fact, it appears from several studies that dynorphin/KOR systems are connected with the dopamine pathway [11]. More specifically, KOR activation is believed to inhibit dopamine release and avoid the hypodopaminergic state responsible for the hypersensitivity to emotional distress during drug withdrawal and abstinence. KOR agonists and drugs of abuse seem to have opposite effects on both abuse related neurochemical and behavioral end points [12]. These effects can be attributed to the ability of KOR agonists to form oligomers with the dopamine transporter (DAT) and decrease dopamine levels in the nucleus accumbens.

In this review, the classic and novel KOR-selective agonists are presented, subdivided among small molecules and peptides, and the pharmacological features of the most interesting examples are discussed in detail. From this recognition, it appears that there are, at present, three main trends in the development of safer analgesics acting at KOR. In the last decade, the most followed strategy was the development of biased agonists, aiming to separate analgesia from the adverse effects [13,14]. A biased agonist is defined as a ligand which is able to stabilize a distinct conformation of the receptor to specifically activate G-protein signaling without recruitment of β-arrestin, since the latter is considered the effector of the side effects. Several new molecules have been identified as G-protein-biased KOR selective agonists, and these molecules showed promising, more favorable therapeutic profiles.

However, there is controversy in this area; a different explanation for the behavior of these agonists revolves around differences in intrinsic activity [15]. In this case, analgesia with low side effects would be the result of reduced efficacy of the agonist, producing amplification of G-protein, but not β-arrestin pathways. As a consequence, partial KOR agonists are currently under investigation for the development of safer analgesics.

Finally, it can be expected that opioid-associated adverse effects and abuse potential would be greatly reduced if drugs could be excluded from reaching the CNS. Generally, peptides show very reduced central bioavailability compared to small-molecules, due to higher molecular weight and scarce lipophilicity. Therefore, what is generally regarded as an Achilles’ heel can be exploited to design peripherally restricted agonists. Following this principle, diverse opioid peptides have been successfully designed to retain analgesic efficacy against visceral pain with a more favorable adverse effect profile [16].

In particular, this review is aimed at discussing the structural determinants at the basis of KOR activation and biased agonism, starting from the analyses of the ligand-receptor complexes as obtained from X-ray or cryoEM, or as estimated by molecular docking.

For a comprehensive survey of the therapeutic applications of KOR agonists, as well as of the induced side effects (reduced or increased), we refer to the very recent, excellent piece of work by Dalefield et al. [9].

## 2. KOR Agonists

In this section, selected examples of molecules with a clear activity at KOR are reviewed. Molecules such as for instance Noribogaine, known as a potent serotonin reuptake inhibitor, which also acts as a moderate KOR agonist and weak MOR agonist or weak partial agonist, are not included.

### 2.1. Small Molecules

#### 2.1.1. Morphinans

Nalfurafine is a selective KOR agonist approved for clinical use as an antipruritic. It is considered a biased agonist because an in vitro experiment resulted in its preferred activation of β-arrestin pathway signaling [17]. Unexpectedly, even if this pathway usually elicits side effects, in vivo it does not appear to be responsible for KOR agonist-induced aversion.

**Figure 1:**
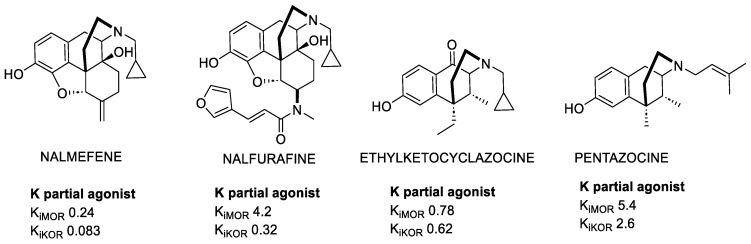
Examples of relevant morphinans and benzomorphans. K_i_ values are also shown (nM).

Nalmefene (NMF) is a partial KOR agonist and potent MOR antagonist (Figure 1) utilized for opioid and alcohol addiction [18], whose most commonly occurring adverse events are nausea, insomnia, and dizziness.

#### 2.1.2. Benzomorphans

The κ-type receptor owes its name to ketocyclazocine, the first selective KOR ligand [19], which elicited analgesia and sedation. Ketocyclazocine belongs to the benzomorphans class, obtained by morphine skeleton reduction. Despite ketocyclazocine resulting to be less potent than morphine, Benzomorphans (Figure 1) were studied for many years for their interesting mixed KOR agonist/MOR antagonist profile. Their clinical development was precluded because they also induce respiratory depression and other side effects.

In contrast, Pentazocine is still under investigation, and recent studies showed that it was more potent in activating p38 MAPK mediated by h-KOR than the rat KOR, and it was also shown to be a potent promoter of β-arrestin 2 recruitment [20]. Studies on Pentazocine led to the identification of Bramazocine as a potent KOR agonist. Despite its activity profile, it does not seem to elicit morphine-like adverse actions, and it can be used for its analgesic and diuretic properties. Nonetheless, its psychotomimetic side effects limit its use as a clinical analgesic, but still allow for its use in the treatment of addictions [21].

#### 2.1.3. Arylacetamides

**Figure 2:**
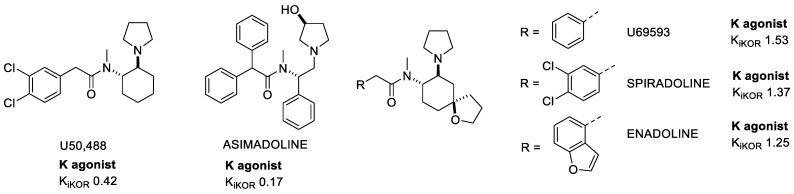
Examples of Arylacetamide KOR agonists. K_i_ values are also shown (nM).

The class of KOR-selective Arylacetamide derivatives (Figure 2) was described for the first time in 1982 [22]. Among them, U50,488 emerged for novel structure, potency, and selectivity, with analgesic, antipruritic, and other effects, Unfortunately, U50,488 and its subsequent derivatives showed all the side effects correlated with the activation of KOR, and also a modest permeability of the blood–brain barrier (BBB).

Asimadoline was found to inhibit nociception via activation of KORs expressed on the peripheral endings of nociceptors in the colon, suggesting a peripherally restricted action which might be useful for a variety of painful conditions in the viscera, such as irritable bowel syndrome (IBS) [23].

With the attempt to further improve the structure of U50,488, a spiroester was added to the cyclohexane ring, leading to a special branch of the Arylacetamide family of which U69593 was the precursor. U69593 produced antinociception without affecting GIT mobility, suggesting a central but not a peripheral activity. Both U50,488 and U69593 activated, with the same efficacy, G-protein and β-arrestin signaling, so they have been classified as full unbiased KOR agonists [24].

Further modifications lead to Spiradoline (U62-066) and Enadoline (CI-977) molecules being capable of crossing the BBB, with reasonable efficacy and with an analgesic potency comparable to morphine, however with reduced respiratory depression. Unfortunately, they still have dose related central effects such as anhedonia, dysphoria, and hallucination-like effects so it was impossible to carry on further clinical investigations [14].

Diverse alkyl- or aryl-substituted ethyl groups were introduced over the years to the base structure in order to achieve higher analgesic efficacy with moderate addictive potential. Among the derivatives it is worth mentioning ICI 199,441, GR89,696, R-84760. Unfortunately, none of these molecules were able to complete the clinical trials due to adverse reactions.

#### 2.1.4. Triazoles

**Figure 3:**
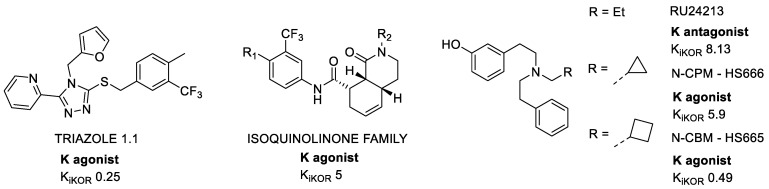
Examples of KOR agonists: triazoles, isoquinolines, and diphenethylamines. K_i_ values are also shown (nM).

As a result of two different screenings, in 2013 Zhou et al. discovered two new classes of KOR selective agonists: triazoles and isoquinolines [25] (Figure 3). Both of them showed high binding affinity with a preferential activation of G-protein and minimal effects on β-arrestin recruitment and downstream ERK1/2 activation. Further studies revealed that triazole, in particular, induced antinociception, without altering locomotion nor provoking dysphoria or aversion. Triazole 1.1 retained the antinociceptive and antipruritic efficacies of conventional KOR agonists, yet it did not induce sedation or reductions in dopamine release in vivo, nor did it produce dysphoria as determined by intracranial self-stimulation in rats [13].

#### 2.1.5. Diphenethylamines

These simple molecules were preliminarily described in 1978 as potential anti-Parkinson’s drugs (Figure 3) [26]. The first synthetized molecule was RU24213, and after it libraries of diverse molecules were designed. The first strategy was the extension of the N-alkyl substituent. In vitro binding studies showed good KOR affinity and potency, but weak selectivity with a decreasing trend as the number of carbons increased [27].

Subsequently, the introduction of bulkier substituents allowed for an increase in KOR affinity, selectivity, agonist potency, and efficacy, giving N-cyclobutylmethyl (N-CBM, HS665) and N-cyclopropylmethyl (N-CPM, HS666) [28]. HS665 and HS666 were shown to produce potent antinociception, mediated by central KORs. In particular, HS666 showed reduced liability for aversive effects, and this was correlated with its lower efficacy in the β-arrestin2 signaling pathway [29].

Considering the inclusion in recent years of fluorine in drug candidates, in 2017 Erli et al. expanded the SAR studies on the original series, e.g., by examining any effects of fluorine at position 2 of diphenethylamines, and by introducing bulkier N-substituents, plus additional hydroxyl groups at positions 3′ and 4′. It was demonstrated that some analogues gained sub-nanomolar affinity and excellent KOR selectivity, acting as full or partial agonists or as antagonists [30].

#### 2.1.6. Salvinorins

**Figure 4:**
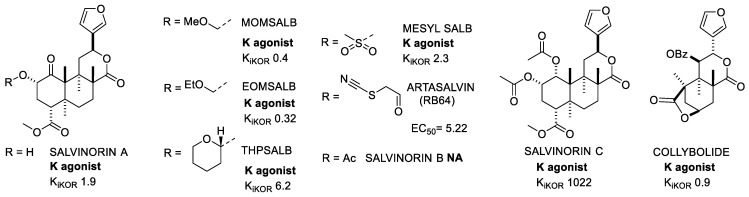
Salvinorin A and selected analogues and derivatives. K_i_ values are also shown (nM).

Salvinorin A (SalA) (Figure 4) is a terpenoid isolated in 1982 by Ortega et al. from Salvia Divinorum, a herbal plant native to the southwestern region of Mexico [31]. Two years later, Valdes et al. extracted Salvinorin B [32] and in 2001 also Salvinorin C [33]. These compounds were found to be selective KOR agonists with analgesic and hallucinogenic effects. All of these compounds immediately gained interest because of their lack of structural similarity to the other psychotomimetic substances. Indeed, unlike the conventional opioid ligands, salvinorins do not contain any nitrogen atoms.

Unfortunately, these natural molecules suffered from a short half-life and rapid onset of action; therefore, a large number of analogues were prepared to improve the pharmacokinetic profile, generally by modifying the positions at C2 and C4 of the furan ring [34]. In some cases, e.g., MOM-SalB, EOM SalB, and THP SalB (Figure 4), modifications gave stronger affinities or potencies and improved metabolic stability compared to SalA, but unfortunately this did not translate to increased brain residence time.

Further studies were performed in vivo on anti-addictive, mood, locomotive, and aversion effects. In particular, in 2013, Morani et al. [35] tested MOMSalB, for cocaine-seeking behavior [36]and the study showed a similar effect, compared to SalA, in reducing cocaine primed-induced reinstatement [35].

Mesylation at C2 gave Mesyl SalB, which showed distinct features and differences with respect to the other analogues. When tested for the inhibition of foskolin-stimulated cAMP accumulation, it showed increased potency in vitro. This in vitro result, together with the similar efficacy to U50488 in the recruitment of β-arrestin, suggested a G-protein biased profile. Furthermore, Mesyl SalB had fewer side effects than SalA and U50488 when tested for cocaine addiction, but it maintained pro-depressive effects and showed no antinociception activity [37].

Additionally, RB64 was identified as a biased G-protein KOR agonist with the specific characteristic of inducing less receptor internalization, having longer lasting effects and less unwanted reactions (anhedonia or motor activity alteration), while still inducing aversion [38].

Collybolide was extracted in 1974 from the fungus Collybia Maculata [39], and attracted attention as a non-nitrogenous, potent, and biased KOR agonist. However, more recent studies indicated that collybolide was mischaracterized as a KOR agonist [40].

### 2.2. Peptides and Peptidomimetics

Dynorphin is one of the endogenous 17-mer neuropeptide ligands of KOR. Other peptides with KOR activity have been identified in fungi and invertebrates, e.g., conotoxins, CJ15,209, and several others have been obtained by chemical synthesis [41]. Native peptides such as dynorphin have the potential to potently activate opioid receptors. However, their potential clinical use is hampered by poor pharmacokinetic properties. As a consequence, starting from the native sequences, a variety of peptidomimetics were designed with the perspective of increasing enzymatic stability and bioavailability [42,43].

#### 2.2.1. Dynorphins

Dynorphin A (DynA)1-17 (YGGFLRRIRPKLKWDNQ), isolated in 1975 from porcine pituitary, is an endogenous neuropeptide capable of modulating pain, addiction, and mood [44]. Several isoforms were soon identified: Dynorphin A(1-8), Dynorphin B(1-13), big Dynorphin, and Leumorphin. Dynorphin A (DynA)(1-17) is regarded as the specific endogenous ligand of KOR [6]. Extensive SAR studies have identified DynA fragments critical to bioactivity. The 17 amino acid full sequence was shortened at its C-terminus to give Dynorphin(1-13), without altering activity. While the N-terminal “message sequence” (YGGF) is essential for binding to all opioid receptors, the C terminal “address sequence” (LRRIRPKLKWDNQ) is required for potency and selectivity. In addition, replacement of the nine C-terminal residues of DynA(1-17) with those of Nociceptin also maintained activity at KOR [45]. From this observation, it was concluded that the essential pharmacophores of DynA(1-17) lie within the N-terminal fragment DynA(1-8) (YGGFLRRI).

#### 2.2.2. Difelikefalin (CR845)

**Figure 5:**
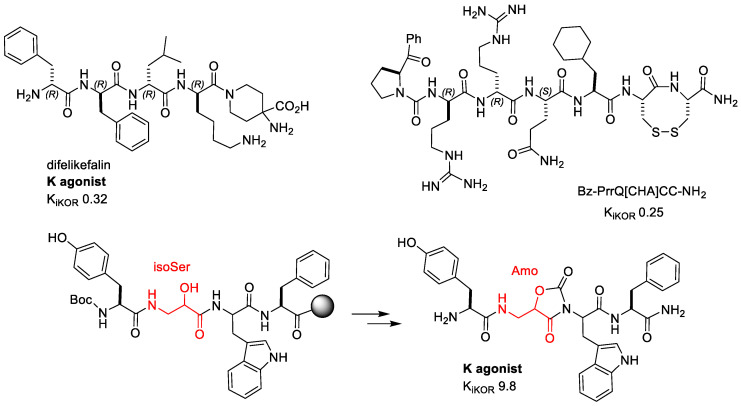
Peptidic and peptidomimetic KOR agonists.

In search of KOR agonists with improved in vivo stability, several research groups designed oligopeptides comprising D-amino acid, and these studies yielded molecules which appeared suitable for assessment of peripherally acting analgesics. Most of these oligopeptides shared a common N-terminal D-Phe-D-Phe [46,47,48,49] including Difelikefalin (CR845) (Figure 5), an all-D-amino acid tetrapeptide developed by Cara Therapeutics with high selectivity for KOR that has been shown to be effective in the treatment of chronic pruritus [50] and post-operative pain after abdominal surgery [51]. Due to its hydrophilic properties, transport across the blood–brain barrier is limited. Although Difelikefalin is devoid of detrimental effects linked to the centrally expressed KOR, its intravenous delivery remains a key obstacle for its widespread use.

#### 2.2.3. Conorphins

Marine cone snails are equipped with a venom apparatus that typically contains 1000 unique venom peptides called Conotoxins or Conopeptides, usually characterized by a S–S bond-cyclic framework. Among these peptides, ω-Conotoxin MVIIA (Ziconotide), an inhibitor of N-type VGCCs, is on the market for the treatment of chronic pain [52]. A new KOR agonist named Conorphin-T (NCCRRQICC) was identified from the screening of a Conus venom peptide library across the opioid receptors. Estensive SAR studies generated a new class of enzymatically stable molecules with exceptional plasma stability, excellent affinity, and high KOR selectivity [53]. The lead NCCRRQICC, which had a short plasma half-life (t1/2 15 min), was systematically altered to produce the octapeptide Bz-PrrQ[CHA]CC-NH_2_ (Figure 5), which showed sub-nM affinity, good efficacy (K_i_ 0.25 nM, EC50 0.36 nM), and improved plasma stability (t1/2 240 min). The Conorphin agonist inhibited nociceptors in the colon, in a mouse tissue model of chronic visceral hypersensitivity, suggesting the potential of KOR agonists for the treatment of chronic abdominal pain [53].

#### 2.2.4. Helianorphins

Gruber et al. utilized cyclic peptides from plants as templates for the design of a stable KOR peptide ligand, acting as a potent analgesic for enteric administration [54]. Specifically, different Dyn(1−13) epitopes were grafted with parts of the peptide SFTI-1, one of the main peptides in the extract of sunflower seeds. SFTI-1 is a bicyclic, “8”-shaped peptide, due to the presence of one disulfide bond, which can be divided in binding and cyclization loops. Dyn(1−13) was incorporated into either the cyclization loop or the binding loop, or it was split into two fragments and inserted into both loops. This strategy allowed for identification of Helianorphin-19, c[CYGGFLRRCIRPKLK], a selective KOR ligand with good affinity (for KOR, K_i_ 21 nM, EC50 45 nM). Helianorphin-19 was found to preferentially activate G-protein over β-arrestin 2, and to be active in a CVH mouse model of abdominal pain; this is possibly correlated to its stability in the GIT tract as estimated in simulated gastric fluid, while it did not affect motor coordination nor induce central analgesia.

#### 2.2.5. H-Tyr-Amo-Trp-PheNH_2_

Bedini et al. synthesized a mini-library of diastereomeric and constrained analogues of the endogenous, highly selective MOR agonist Endomorphin-1 (EM1), H-Tyr-Pro-Trp-PheNH_2_ [55,56,57] by introducing β^2^-homo-Freidinger lactam-like scaffolds at position 2 [58] (Figure 5). The 5-(aminomethyl) oxazolidine-2,4-dione (Amo) scaffolds were obtained by in-peptide cyclization of isoserine-Trp [59]. Intriguingly, the all-(S) configured H-Tyr-Amo-Trp-PheNH_2_ displayed high KOR affinity (K_i_ 9.8 nM) and high selectivity. The peptidomimetic inhibited forskolin-induced cAMP accumulation as a partial agonist, not activating β-arrestin signaling. In the tail-immersion test, the peptidomimetic was determined to have a relevant analgesic effect (20 mg/kg, ip: 60% MPE at 15 min, and still 42% MPE at 30 min), and the effects were counteracted by the preemptive administration of the KOR selective antagonist nor-BNI, thus confirming, also in vivo, a KOR-mediated activity.

#### 2.2.6. CJ-15,209 and Derivatives

**Figure 6:**
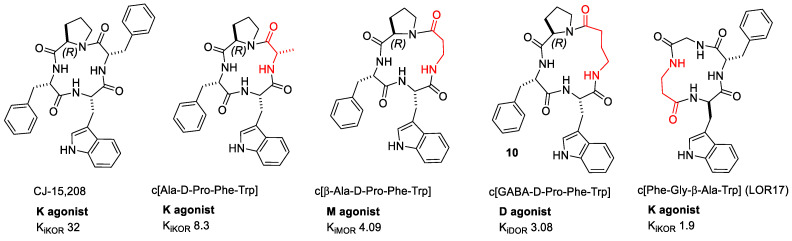
CJ-15,208 and some relevant derivatives. K_i_ values are also shown (nM).

In 2004, Saito et al. isolated from the fermentation broth of the fungus Ctenomyces serratus ATCC15502 the cyclotetrapeptide c[Phe-D-Pro-Phe-Trp] (CJ-15,208) (Figure 6). CJ-15,208 was a modestly selective KOR/MOR ligand (IC_50_ nM: KOR, 47; MOR, 260 DOR, 2600). Initially, the researchers observed antagonist activity against the KOR agonist asimadoline in the rabbit vas deferens smooth muscle assay (EC50 1300 nM) [60]. Later, the antagonism was confirmed by the [^32^S]GTPγS functional test [61]. An Ala-scan highlighted the importance of the residues Phe^3^ and Trp^4^; indeed, c[Ala-D-Pro-Phe-Trp] (Figure 6) showed low nanomolar affinity for KOR and MOR, while the other Ala derivatives suffered a substantial loss in binding affinity [62].

The reversal of the configuration at Trp gave c[Phe-D-Pro-Phe-D-Trp], a dual KOR/MOR antagonist in the [32S]GTPγS test (IC50 140 nM) [61]. Additionally, in this case, Ala-scan was not tolerated at positions 3 and 4, since c[Ala-D-Pro-Phe-D-Trp] was the only compound equipotent to the parent (Figure 6) [61]. All of the derivatives of CJ-15,208 maintained the same mixed KOR > MOR affinity profile, albeit with different K_i_ values, and did not exhibit any agonist activity in vitro.

Unexpectedly, in vivo tests showed contrasting activities when compared to the parent peptides. As expected, c[Phe-D-Pro-Phe-D-Trp] behaved as a KOR antagonist also in vivo and prevented the stress-induced reinstatement of extinguished cocaine-seeking behavior [63]. In contrast, the natural isomer CJ-15,208 exhibited robust antinociceptive activity in the warm-water tail withdrawal test, following icv administration [63]. Intriguingly, the Ala analog c[Ala-D-Pro-Phe-D-Trp] also produced potent OR-mediated antinociception in vivo [64]. Finally, CJ-15,208 and c[Phe-D-Pro-Phe-D-Trp] [65,66] and other phenylalanine stereoisomers [67] were found to be orally active and appeared to penetrate the CNS.

The structures of CJ-15,208 and all its derivatives appear clearly correlated to that of c[Phe-Gly-Tyr-D-Pro-D-Trp] [68], a cyclopentapeptide (CPP) discovered independently from CJ-15,208, designed as a cyclic analogue of the EM1. The CPP was found to be a MOR ligand (K_i_ 10^−8^ M), partial agonist in the cAMP functional assay. After systemic administration, it produced antinociception in a mouse model of visceral pain, while the parent EM1 was completely ineffective [69]. Subsequent modifications gave c[Phe-Gly-Tyr-Gly-D-Trp] and c[Phe-D-isoAsp-β-Ala-D-Trp], which showed 10-fold improved MOR affinity, while maintaining the agonist profile [70]. Reduction in molecular complexity yielded the MOR-selective agonist tripeptide Ac-D-Trp-Phe-GlyNH_2_ [71]. The introduction of different substituents at the indole of D-Trp influenced BBB permeability, allowing a measurable MOR-mediated central antinociception in the mouse warm-water tail withdrawal test after ip administration [72].

These Trp-containing macrocycles are clearly an alternative, with respect to the classic opioid peptides, as they lack the protonable amino group of Tyr^1^, commonly regarded as the fundamental “message” pharmacophore [73,74]. Despite the close structural similarities, the two families of opioid peptides showed distinct receptor selectivity and in vivo activity. This led us to presume a correlation between bioactivity and 3D displays of the pharmacophores, which depend in turn on ring size, stereochemistry, and secondary structures.

This hypothesis led to expansion of the ring of CJ-15,208 to the 13-membered c[β-Ala-D-Pro-Phe-Trp], which turned out to be a highly selective MOR ligand (K_i_ 4.1 nM). Surprisingly, a further enlargement of ring size by introducing GABA at position 1 produced a different receptor selectivity. The 14-membered c[GABA-D-Pro-Phe-Trp] (GABA, γ-aminobutyric acid acid), showed very scarce MOR affinity and gained high DOR affinity (K_i_ 3.08 nM) [75]. c[β-Ala-D-Pro-Phe-Trp] inhibited forskolin-induced cAMP production (IC_50_ 6.1 nM and Emax 90%), suggestive of full agonism, while the DOR≫MOR ligand c[GABA-D-Pro-Phe-Trp] inhibited forskolin-induced cAMP accumulation in HEK/MOR (IC_50_ 183 nM). Interestingly, the latter did not alter forskolin-induced cAMP accumulation in HEK/DOR, but significantly antagonized, in a concentration-related manner, the inhibition of forskolin induced cAMP accumulation by 10 μM DPDPE (IC_50_ 7.4 nM). In a mouse model of visceral pain, c[β-Ala-D-Pro-Phe-Trp] elicited peripheral preemptive antinociception (0.5–10 mg/kg, ip: ED50 0.64 mg/kg), and the effect was prevented by pretreating the animals with the antagonist NLX—but not by the DOR selective antagonist NTD, or by the KOR selective antagonist—thus confirming that the observed effect was MOR-dependent.

More recently, the same authors designed the minimalist CJ-15,208 analogue c[Phe-Gly-β-Ala-D-Trp] (LOR17). The peptide was a KOR selective agonist in different cell models with nM affinity (_Ki_ 1.19 nM) [76]. In contrast to U50,488, LOR17 displayed functional selectivity toward G-protein signaling and provoked antinociceptive/antihypersensitivity effects in different in vivo models, including neuropathy by oxaliplatin.

The peptides described in this paragraph constitute a distinct family of tryptophan-containing non cationizable opioid peptides. In contrast to most opioids, their atypical bioactivity appears to reside in the minimal pharmacophoric motif Trp-Phe, with indole being fundamental to the ligand-receptor interaction. The diverse preferences for MOR, DOR, and KOR, seem to depend on specific secondary structures [77].

## 3. Recent Paradigms in KOR Agonism: Biased, Partial, Peripheral

### 3.1. Biased Ligands

The term biased signaling refers to the activation of well-defined signaling pathways over unwanted ones, and in the last decade, this has been the leading trend in the OR opioid field. The hypothesis is that, upon stabilizing distinct receptor conformations, ligands capable of activating G-protein-dependent signaling over β-arrestin dependent pathways might facilitate the development of benign drugs [78]. Nonetheless, the potential utility of functionally selective agonists at ORs is highly controversial.

The first G-protein-biased opioid ligand reported in the literature was the MOR ligand (R)-TRV130 (oliceridine). This compound caused only limited β-arrestin recruitment and receptor internalization [79]. TRV130 seemed to elicit potent antinociception in vivo, without inhibiting GIT transit or causing respiratory depression [80]. After TRV130, other Gi-protein biased agonists have been identified. The MOR ligand PZM21 was discovered by in silico screening [81] and was proposed as an analgesic with an unprecedented profile and reduced side effects in the hot-plate assay, but not in the tail-flick test.

Despite the promising preclinical in vivo data, TRV130 failed in clinical trials [82] due to side effects comparable to that of morphine and other opioids, i.e., nausea, vomiting, dizziness, headache, and constipation. Recently, it was reconsidered and approved for moderate and severe pain in adults by intravenous administration [83]. Contradictory results were reported also for PZM21 [84], which was reclassified as a low efficacy MOR unbiased agonist (i.e., activating both G-protein and β-arrestin 2 signaling), which depressed respiration and induced tolerance.

Interestingly, experiments with a non-phosphorylatable version of MOR knocked into mice [85] showed that total abolishment of β-arrestin binding improved analgesia and reduced tolerance but worsened other opioid side effects. These new data supported that respiratory depression and constipation are driven by G-protein-mediated signaling, not by β-arrestin [86]. Plausibly, the reduced conditioned place preference of PZM21 was due to the potent antagonist activity of PZM21 at the KOR, which has been shown to have significant influences on reward processing.

These failures led to much debate about the practical utility of biased agonism at MOR. Several researchers suggested that the separation between analgesic effects and unwanted side effects may not be simply due to G-protein versus β-arrestin-mediated effects [87,88].

#### Biased Ligands at KOR

**Figure 7:**
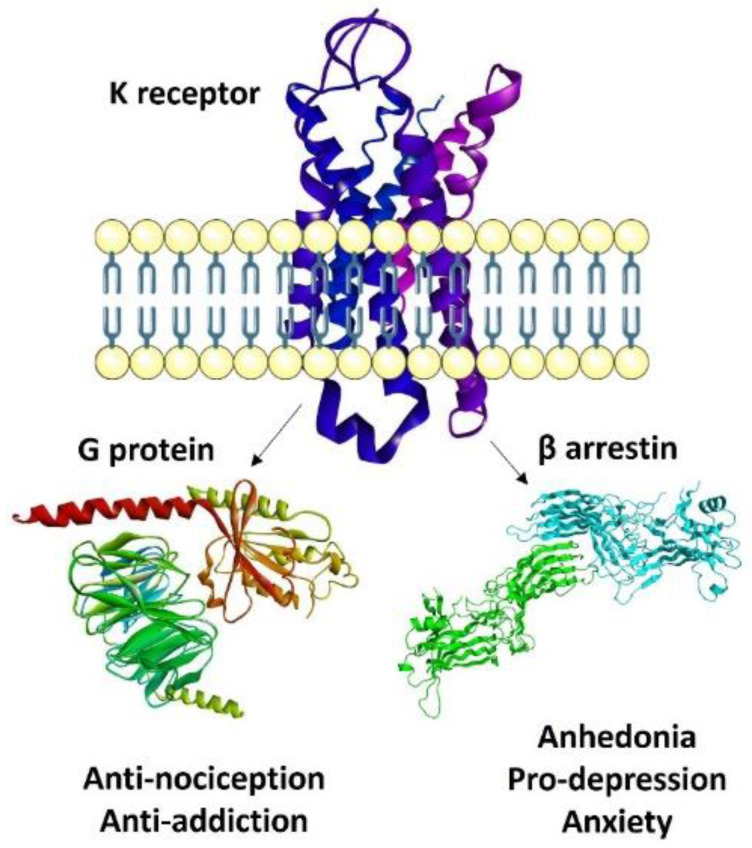
Activation and linked effects of G-protein and arrestin.

Although biased agonists at MOR have failed so far as therapeutics in the market, functional selectivity at KOR is still considered interesting and potentially promising. There is evidence that KOR signaling through G-protein pathways (including adenylyl cyclase inhibition and early ERK1/2 phosphorylation) mediates the antinociceptive and anti-pruritic effects of KOR agonists, whereas β-arrestin-2-dependent signaling (including p38MAPK activation) mediates the dysphoric effects as well as sedation, and motor incoordination [13,89] (Figure 7). Some prototypic, interesting studies are discussed here.

The 1-pyrazole methyl ester MPCI was shown to elicit KOR-mediated antinociception without sedation, constipation, or motor impairment in different models of inflammatory and neuropathic pain [90]. Zhou et al. described two classes of G-protein-biased KOR agonists with only weak recruitment of β-arrestin-2. The prototypic triazole 1.1 and isoquinolinone 2.1 displayed antinociceptive effects in the warmwater tail-flick assay, similar to U50,488 [25]. Recently, triazole 1.1 was shown to induce dose-dependent, KOR-mediated antinociception, and it was assayed in an in vivo model of pruritus without affecting ambulatory behaviors in mice [38].

In search for new KOR ligands, Zheng et al. conducted a virtual screening of available compound libraries and identified compound 81 as an agonist with a G-protein biased profile [91]. Antinociception devoid of dysphoria promoted by a KOR-selective, G-protein-biased agonist, was observed also by Spetea et al. [28].

On the other hand, White and co-workers reported that the G-protein-biased KOR agonist 22-thiocyanatosalvinorin A (RB64) induces antinociceptive effects in the hot-plate test without impairing rotarod performance or novelty-induced locomotion, and did not increase anhedonia. Surprisingly, RB64 is aversive in the conditioned place preference test, albeit not involving KOR mediated recruitment of β-arrestin-2 [38].

Recently, three phenethylamine compounds showed full KOR agonism, and one of these compounds was clearly G-protein biased, while the others showed different degrees of functional selectivity. As expected, only the latter produced rotarod incoordination [30].

6′-Guanidinyl-naltrindole (6′-GNTI) is a potent analgesic in several assays that does not induce conditioned place aversion, nor does it influence motor activity. Recent studies suggested that 6′-GNTI acts as a G-protein partial agonist of the KOR at low μM concentrations and does not activate the β-arrestin 2 pathway in the sub-nM range [92].

The Northebaine derivative SLL-627 has been identified as a highly selective and potent KOR full agonist in vitro and in vivo. Besides having low liability to the conditioned place aversion (CPA) test, treatment with SLL-627 did not reduce locomotor activity compared to most of the other KOR agonists which generally exhibited sedative effects [93].

The cyclotetrapeptide LOR17, c[Phe-Gly-β-Ala-D-Trp], lacking any protonable amino group, was designed as an analogue of CJ-15.208. LOR17 was a potent and G-protein biased KOR agonist with a more favorable in vitro and in vivo pharmacological profile compared to U50,488. Indeed, LOR17 inhibited adenylyl cyclase similarly to U50,488, suggesting full agonism, but did not significantly recruit β-arrestin 2 at KOR, (bias factor G-protein activation/β-arrestin 2 recruitment = 853). Consistently, LOR17 activated early ERK1/2 phosphorylation in different cell models, whereas contrary to U50,488, it did not activate p38MAPK and does not promote β-arrestin 2-dependent, p38MAPK-mediated astrocyte proliferation. As for U50,488, LOR17 was effective in animal models of acute nociception, and reduced thermal hypersensitivity in a mouse model of neuropathic pain induced by oxaliplatin after both single or repeated s.c. administrations, without altering motor coordination, locomotor, and exploratory activities and also without inducing pro-depressant effects [76].

The data discussed above support that downstream of KOR activation, β-arrestin-2 recruitment may stimulate sedation, coordination impairment and anhedonia, and arrestin-independent p38MAPK activation may possibly produce aversion, at least in rodents. In this perspective, functionally selective KOR agonists may activate G-protein-mediated signaling to produce antinociception, over β-arrestin 2-dependent induction of p38MAPK, which preferentially contributes to adverse effects.

### 3.2. KOR-Selective Partial Agonists

In the preceding paragraph, biased agonism has been invoked to explain the alternative pharmacological profiles. However, these differences might depend also on drug efficacy, and not only on signaling bias. Hence, the above mentioned differential signaling profiles could also be explained in terms of low efficacy, stimulus amplification, and receptor reserve [87]. As a matter of fact, many compounds regarded as KOR biased and characterized by good therapeutic indexes are indeed partial agonists, whose efficacy can be likely attributed to low efficacy partial agonism rather than G-protein-bias [15].

A good example of this concept is furnished by HS666. This diphenethylamine derivative showed a reduced liability for aversive effects, and this was correlated with its low efficacy in the β-arrestin2 signaling pathway. However, HS666 is also a partial agonist, as determined by the [^35^S]-GTPγS binding assay (Emax 50%) and is further confirmed by its partial inhibition of U69,593-stimulated G-protein coupling, as compared to full antagonism produced by nor-BNI [29].

In other words, KOR partial agonists may allow an analgesic response to be produced at dosages lower than those required to produce the adverse effects. A G-protein-dependent output (e.g., inhibition of cAMP accumulation) encompasses high amplification, while arrestin recruitment at the receptor has little or no amplification. This may lead to definitions of “biased” ligands that are not biased at all.

Partial agonists may hold potential for the treatment of depression, mood disorders, psychiatric comorbidities, and specific drug addictions. However, some partial KOR agonists display anti-inflammatory and neuroprotective effects, and they suppress the rewarding effects of opioids and cocaine. Such compounds may restore homeostatic control of dopaminergic function underlying mood and reward. KOR partial agonism may also diminish the severity of relapse/re-escalation. Selective KOR partial agonists may be beneficial in promoting more prolonged abstinence, as well as decreasing the severity of relapse episodes. Mixed agonists/partial agonists/antagonists at different OR subtypes are employed to treat alcohol dependence and cocaine craving [94]. As for KOR antagonists, these are already used to treat opioid dependence and withdrawal [95,96]. Examples of partial KOR agonists have been provided in the preceding sections.

In summary, the clinical utility of KOR ligands with partial efficacy has been well documented. Unfortunately, owing to lack of KOR>MOR selectivity in known ligands, these therapeutic opportunities have not been clinically exploited so far. In this respect, it is worth mentioning the peptide Tyr-Amo-Trp-PheNH_2_. This peptide was a selective ligand of KOR with nanomolar affinity, and behaved as a partial agonist in the functional test, since it inhibited forskolin-induced cAMP accumulation in HEK cells stably expressing hKOR, with IC_50_ 0.22 nM and Emax 40%, compared to U50,488 (IC_50_ 1.2 nM and E*_max_* 90%). When the peptide (1 μM) was co-administered together with U50,488, a 40% inhibition of forskolin-induced cAMP accumulation was observed and the U50,488 concentration–response curve was shifted rightward, thus, confirming a partial agonist activity for the peptide [58].

### 3.3. Central vs. Peripheral Activity

Unlike MOR agonists, KOR agonists do not induce euphoria/addiction, respiratory depression, or GIT transit inhibition. Therefore, they were initially viewed as an attractive alternative strategy to design potent and safer analgesics. Spiradoline and Enadoline, and other small-molecule KOR agonists of the first generation, were orally active, brain penetrating, and lacked morphine-like side effects. However, these molecules were associated with unwanted neuropsychiatric effects, such as sedation and dysphoria. Peripheral KORs are also able to induce analgesia, particularly after tissue injury and inflammation. Therefore, subsequent studies have been conducted on achieving analgesia while avoiding CNS penetration, in order to try and not trigger the side effects.

Asimadoline was the first molecule to enter clinical trials with the hopes that it might treat peripheral pain. In fact, it appears to inhibit nociception via activation of KORs expressed on the peripheral endings of colonic nociceptors, suggesting a peripherally restricted KOR agonist might be a useful treatment for a variety of visceral pain conditions including irritable bowel syndrome (IBS) [23]. Unfortunately, asimadoline still produced central adverse effects. However, asimadoline induced hyperalgesia in non-visceral postoperative pain.

A simple way to reduce central effects is to decrease the ability of KOR agonists to penetrate across the BBB [97]. This is typically carried out by performing structural modifications to reduce lipophilicity. Another strategy is to turn to peptide-based compounds. Indeed, peptides are known to have limited ability to cross the BBB [98]. This opportunity yielded peptidic KOR ligands, such as the all-D configured sequence Difelikefalin (CR845) [50,51]. Compared to many other opioids, Difelikefalin exhibits a minimal effect on the CNS and does not cause respiratory depression or sedation, somnolence or paresthesia, nor any euphoric effects. In 2021, Difelikefalin was approved by the Food and Drug Administration (FDA) and in 2022 by the European Medicines Agency (EMA) as the first drug for the treatment of CKD-aP in adult, hemodialysis patients.

KOR agonists are particularly attractive as analgesics for visceral pain [99]. Helianorphin-19 was effective in a CVH mouse model of abdominal pain, possibly correlated to its stability in the GI tract, as estimated in simulated gastric fluid, while it did not affect motor coordination, nor did it induce central analgesia [54]. The conorphin-derived agonist Bz-PrrQ[CHA]CC-NH_2_ (Figure 5) was a potent (K_i_ 0.25 nM, EC50 0.36 nM) and selective KOR agonist with excellent plasma stability (t1/2 240 min). This peptidomimetic inhibited colonic nociceptors in a mouse tissue model of chronic visceral hypersensitivity, suggesting the use of KOR agonists for the treatment of chronic abdominal pain [53].

It must be mentioned that, in contrast to linear peptides, cyclic peptides might show some ability to cross the BBB [42,43]. For instance, the macrolactam peptide LOR17 was a full KOR agonist with functional selectivity toward G-protein-coupled intracellular signaling, over β-arrestin-2-mediated pathways. LOR17 was determined to have significant antinociception, both in the acetic acid-induced writhing assay and in the warm water tail-withdrawal assay, when administered in the same range of doses. Thus, this suggests that LOR17 may distribute to the CNS [76].

## 4. Structural Insights in Ligand-Receptor Interactions for KOR Agonists

MOR, DOR, and KOR exhibit remarkably conserved amino acid sequences, with a 70% sequence identity in their seven transmembrane domains, particularly in the orthosteric binding pocket. However, KOR is different from the other opioid receptors in terms of tissue expression patterns, functional properties, and side effect profile upon activation. A detailed analysis of the inactive and active-state structures revealed structural determinants that can be exploited for specificity [100]. In particular, the KOR binding pocket is comparatively much narrower and deeper and partially capped by the ECL2 β-hairpin, while the region between TM2-TM3 of KOR is more hydrophobic and has a number of non-conserved “address” residues such as 6.58 and 7.35 (Ballesteros–Weinstein nomenclature) [101].

### 4.1. Crystal Structure of KOR–JDTic

**Figure 8:**
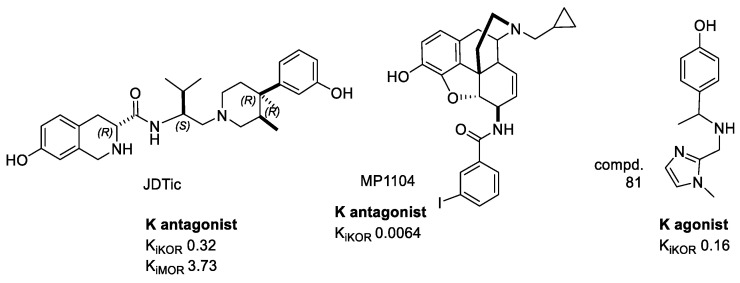
Examples of selected KOR ligands of interest for structural studies: the antagonist JDTic and the agonists MP1104 and compd. 81. K_i_ values are also shown (nM).

**Figure 9:**
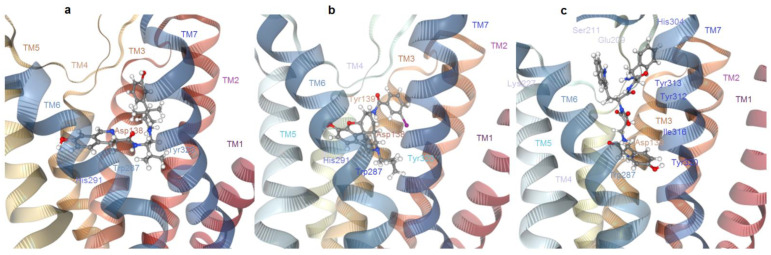
Comparison of the ligand-receptor complexes as extracted from (**a**) the X-ray crystal structures of antagonist JDTic–KOR (inactive), and (**b**) of agonist MP1104–KOR (active). (**c**) Docked structure of Tyr-Amo-Trp-PheNH_2_-KOR (active). Figure obtained with PacDOCK web server [103].

A major breakthrough in the GPCRs field was the disclosure of the crystal structure of a truncated version of human KOR, in complex with the selective antagonist JDTic (Figure 8) at 2.9 Å resolution. T4L phage lysozyme was inserted to replace the highly mobile intracellular loop 3 (ICL3), hence stabilizing the overall KOR structure [102]. The receptor was crystallized in a cholesterol-doped monoolein lipidic cubic mesophase. The JDTic–KOR complex shows the ligand in a V-shaped conformation in the bottom of the binding cleft (Figure 9), stabilized by salt bridges, polar, and hydrophobic interactions with the receptor. The protonated amines in both piperidine and isoquinoline moieties of the ligand form ionic bonds with Asp138(3.32) carboxylate (in brackets, the Ballesteros–Weinstein nomenclature).

The hydroxy group at position 6 of the isoquinoline scaffold forms a hydrogen bond with conserved crystallographic water, mediating an interaction with H291(6.52), while the other phenoxy group interacts with structured water, mediating a contact with V118(2.63). However, JDTic interacts with four residues in the binding pocket that differ from other closely related ORs, i.e., Val 108(2.53), Val 118(2.63), Ile 294(6.55), and Tyr 312(7.35).

Very recently, another structure of inactive KOR–JDTic was solved in a complex with Nb6 nanobody, selectively stabilizing the inactive state of the receptor. However, this work mainly focused on demonstrating the new mode of nanobody binding [104].

### 4.2. Crystal Structure of KOR-MP1104

In 2018, information on KOR was greatly expanded by the determination of an active-state crystal structure of KOR in complex with the potent morphinan agonist MP1104 (Figure 8), and the nanobody Nb39 [105]. The active structure revealed a number of important selectivity features. The shape of MP1104 partially overlaps with the isoquinoline moiety of JDTic (Figure 9), and also forms a similar salt-bridge to the Asp138(3.32). The MP1104–KOR complex is characterized by a ~10% contraction in the volume of the orthosteric site as compared to antagonist-bound inactive state, and a slightly deeper binding of the agonist ligand, likely connected to conformational changes in Met142(3.36) and Trp287(6.48).

Similarly, to other GPCRs, the active structure of KOR differs from the inactive one by a ~10 Å outward movement in the intracellular end of TM6 and a ~3 Å inward movement of TM7. The structure of the complex was utilized for molecular docking of several other important KOR ligands sharing the epoxymorphinan scaffold, including guanidinonaltrindoles (5′-GNTI, 6′-GNTI), and all compounds maintained a similar binding pose.

### 4.3. Docking Simulations

A deep knowledge of KOR specificities is fundamental for the identification and development of subtype-selective agonists and antagonists. Starting from the inactive and active models of the receptor, several research groups have envisaged in silico virtual ligand screening to identify novel lead compounds [106,107,108,109,110]. In general, the effort led to the identification of active KOR ligands characterized by high diversity of bioactive poses among the diverse ligands. In the following sections, some enlightening examples are discussed.

Zheng et al. exploited the crystal structure of JDTic–KOR for a virtual screening campaign aimed at identifying new chemotypes of KOR ligands. After optimization of the receptor model, the screening of millions of commercially available compounds gave a bunch of hits in the sub-micromolar affinity range, including compd. 81 (Figure 8)—a potent Gi biased agonist for KOR with minimal β-arrestin recruitment [91].

Guerrieri et al. performed docking studies for diphenethylamines, and the simulations predicted that the interaction of the ligand with the hydrophobic pocket formed by Val108, Ile316, and Tyr320 influenced ligand binding. Moreover, the phenolic 3-OH group allowed the stabilizing interaction with His291. Bulky N-substituents increased selectivity and affinity, and the introduction of a second hydroxyl group in position 3′ resulted in the identification of potent KOR partial agonists [111].

#### 4.3.1. Docking of Salvinorins

SalA is unique compared to other KOR ligands in that it lacks a charged or polar nitrogen atom to anchor it in the binding pocket. Extensive site-directed mutagenesis, substituted cysteine-accessibility mutagenesis (SCAM), and SAR studies on SalA and its analogues, suggested a variety of possible modes of interaction. After having described the crystal structure of the JDTic-h- KOR [102], the same authors simulated interactions between the cysteine-reactive agonist 22-thiocyanatosalvinorin A, and the receptor. As the thiocyanate group contains two electrophilic centers, this SalA analogue formed two alternative covalent adducts upon binding to Cys315(7.38). Docking analyses revealed very similar binding modes for both adducts. Not unexpectedly, while being an important anchor of binding for many opioids, the interaction with Asp138(3.32) was less critical for salvinorins. In any case, it must be pointed out that alternative docking analyses of diverse salvinorin derivatives gave alternative predicted poses, suggesting the potential existence of a plurality of binding modes.

#### 4.3.2. Docking of Dynorphin

Seminal work in the 1970s and 1980s led to a model of KOR activation by Dynorphin that proceeds via a multistep mechanism. On one side, the N-terminal YGGF “message” sequence, which is common to all opioid peptides, was found to be responsible for receptor activation. On the other side, a C-terminal “address” sequence was considered responsible for KOR subtype specificity through electrostatic interactions. Further molecular docking simulations of DynA(1-8) binding to KOR revealed a partially stabilized α-helix from Phe^4^ to Ile^8^, allowing the YGGF message domain to bind in a hydrophobic binding pocket composed by TM 3, 5, 6, and 7 [112].

The X-ray structure of JDTic–KOR was used to confirm earlier docking results and was supported by receptor mutagenesis and functional studies [113]. The consensus view is that the YGGF message domain interacts with KOR in a similar way to the antagonist JDTic, showing the Tyr^1^-amine residue, involved in a salt bridge with Asp138(3.32) carboxylate, encompassing a conserved hydrophobic pocket. The DynA(1-8)/KOR address domain LRRI adopts an extended conformation, with a Gln115(2.60) to NH interaction at Leu5 being of particular importance, and a prominent salt bridge between Glu297(6.58) and Arg^7^.

The structure of the Dynorphin(1-13) peptide bound to KOR was investigated also by liquid-state NMR spectroscopy, including transferred NOE, using (^15^N-^13^C)–labeled ligand, and by molecular dynamics simulations. These structures’ observations were fundamental, together with dynamics, for a better understanding of the starting poses of Dynorphin for MD. These studies provided quantitative data on a KOR-bound conformation [114].

This work revealed a central helical turn bound on both sides by flexibly disordered peptide segments. This mobility was entirely unexpected for the crucial first four residues, YGGF. The authors identified the pose as typical of an inactive state and a second pose which was attributed to an activated state. In this “active” conformation, the N terminus of Dynorphin forms polar interactions with Asn(3.35) and Asp(3.32) side chains, whereas the Tyr^1^ phenol ring is involved in a π-stacking interaction with Trp(6.48), and an H-bond with Asn(7.45).

#### 4.3.3. Docking of Conorphin

Similarly to the case of the Salvinorins, the lack of any protonable amino group in the Conotoxin derivative Bz-PrrQ[CHA]CC-NH_2_ [53] made docking analysis particularly intriguing. In the best simulated pose, the C=O of the benzoyl residue was predicted to form a strong hydrogen bond with Asp138, positioning the aromatic benzoyl moiety to form hydrophobic interactions with residues of TM3. The guanyl-NH of Arg^3^ forms strong H-bond interactions with Tyr312 and Tyr320 in TM7. Arg^4^ was rotated in the opposite direction forming a salt bridge with Asp223(TM5) and a H-bond with Tyr219(TM5). The amide bond between Arg^4^ and Gln^5^ forms strong hydrogen bonds with Ser211 and Cys210 in ECL2, which provides a binding conformation specific to this peptide. The hydrophobic amino acid CHA at position 5 produces a gain of activity due to hydrophobic interaction with residues of TM7, including a strong interaction with Tyr312. The C-terminal amide of the peptide ligand and Glu209 form a characteristic H-bond interaction with the vicinal disulfide moiety of importance for the placement of the C-terminal amide function. Additionally, the vicinal disulfide moiety also forms van der Waals and hydrophobic interactions with residues at the extracellular parts of TM3, TM4, TM5.

#### 4.3.4. Docking of Tyr-Amo-Trp-PheNH_2_

De Marco et al. designed an EM1 analogue comprising the central scaffold Amo in place of Pro2. This compound represented the first close analogue of EM1 showing nM KOR affinity and complete KOR selectivity, acting as a partial agonist in vitro and showing relevant analgesia in vivo in the tail immersion test. Molecular modeling and docking analysis were utilized to shed light on the bioactive structure of this unprecedented chemotype of KOR ligand [58]. Molecular docking computations in the X-ray structure of the active conformation of KOR (PDB ID: 6B73) supported the role of Amo in orienting the pharmacophores for optimal receptor fitting. Together with the C-terminal portion, the scaffold itself appeared to belong to the “address” of the ligand, being responsible for interactions with residues which are not conserved across the other ORs (Figure 9).

The protonated amine of Tyr^1^ forms the salt bridge with Asp138(3.32) carboxylate, while the phenolic side chain shows interactions with Tyr320 and Trp287. Albeit not totally unusual for KOR ligands [115], Tyr^1^ appeared to adopt a disposition alternative to that of the tetrahydroisoquinoline ring of JDTic [102], and to the docked pose of the Tyr of DynA(1-8) [113,114], but not to the tyramine portion of MP1104 [105], while displaying something in common with the binding pose of the Conorphins [53].

### 4.4. Design of Biased KOR Ligands

In contrast to MOR, for which many studies have been conducted, including conformational analyses, NMR spectroscopy, molecular docking, and molecular dynamics simulation, a general model that can explain biased (i.e., functional) selectivity of signaling pathways for KOR is not available yet [116,117,118]. The studies performed for MOR predicted that different ligands may form alternative complexes with MOR, engaging in specific ligand-receptor contacts. This induces different displays of the cytosolic side of the seven-helices bundle, in particular by stabilizing different angulations of TM6 which seems to favor intracellular coupling to either G-protein or β-arrestin.

As for KOR, starting from the ligand-receptor models discussed above, some research groups were able to identify structural specificities to rationally design functionally selective KOR agonists. Very recently, Uprety et al. studied the biased behavior at MOR and KOR of diverse analogues of the unbiased morphinan MP1104 by means of a combination of SAR studies, receptor mutagenesis, and molecular docking.

These studies led to the identification of subpockets of the orthosteric site as the hot spots for functional selectivity. Ligands capable of placing morphinan-amidophenyl “address” in contact with the TM2-TM3 transmembrane domains were shown to maintain balanced G-protein and arrestin signaling, while morphinan derivatives directing the amidophenyl group towards the TM5-ECL2 region specifically led to the recruitment of G-protein [119]. The differences between KOR and MOR were explained in terms of the different compositions of their TM2/TM3 sub-pockets, which accommodate the hydrophobic amidophenyl arm of the ligands. This sub-pocket is more hydrophobic in KOR due to the presence of the non-conserved V118(2.63) residue, and a conformational change in the conserved Q115(2.60) residue.

Based on the above analysis, the authors proposed that a rational design of MP1204 analogs, which are G-protein biased at MOR or KOR, would require a switch of the amidophenyl arm from the TM2-TM3 sub-pocket to the TM5-ECL2 one for both receptors. To verify this conjecture, polar or charged substituents were introduced at the amidophenyl to disfavor the interactions at the hydrophobic TM2-TM3 pocket of KOR, while basic moieties were introduced for improving the interactions toward the TM5-ECL2 pocket, since the latter includes acidic residues Asp223(5.35) and Glu209(ECL2).

This model was corroborated by the observation that the docking pose in KOR of another G-protein biased ligand, i.e., 6′-Guanidinonaltrindole (6′-GNTI), also showed the guanidino group engaging with a region between TM5 and ECl2, in particular with the residues Asp223 and Glu209 [119].

The conclusions of the previous study drawn for MP1104, and analogues, were at least in part confirmed by the docking analyses of Tyr-Amo-Trp-PheNH_2_ in KOR. This peptide was a G-protein biased, partial agonist [59], and in the bioactive conformation, the C-terminal address section of the peptide made interactions with residues of EL2 (Glu209, Ser211, Leu212), and of TM7 (Tyr313, Tyr312), with His304(EL3), and with Lys227(TM5), while having no interaction with TM2-TM3.

## 5. Conclusions

Due to the implications of KOR activation, KOR agonists have attracted recent attention for their ability to exert potent analgesic effects, without the unwanted side effects typically associated with MOR agonists. However, the development of KOR agonists has also been limited due to untoward CNS-mediated side-effects, such as dysphoria, sedation, and hallucinations. In this review, the most representative classes of KOR agonists have been discussed, and from the survey of the data reported in the literature it appears that three main paradigms have been adopted in the design of safer analgesics: biased, partial, or peripherally acting agonists. Actually, these definitions can overlap, in the sense that a biased agonist can be also a partial agonist, and/or its in vivo distribution can be excluded from the CNS, thus being limited to the periphery. In general, it is still unclear if analgesia mediated by KOR with reduced-side-effect profiles has to be ascribed to biased agonism, or rather to partial agonism. However, the rational drug design of biased ligands is still in its infancy. In particular, with very few exceptions, limited studies have been conducted so far that have depicted a general model of functional selectivity at KOR [14]. To the best of our current knowledge, biased agonists are supposed to activate specific signaling pathways by interaction with specificity subpockets of the orthosteric binding site as the key spots for triggering functional selectivity. Hence, the identification of structural specificities to rationally design functionally selective KOR agonists can be pursued by of a combination of SAR studies and computer assisted design.

## Data Availability

Not applicable.
